# Stress mediates the relationship between sexual orientation and behavioral risk disparities

**DOI:** 10.1186/1471-2458-14-401

**Published:** 2014-04-26

**Authors:** Jennifer M Jabson, Grant W Farmer, Deborah J Bowen

**Affiliations:** 1 Department of Public Health, University of Tennessee, 390 HPER 1914 Andy Holt Ave, Knoxville, TN 37996, USA; 2 Washington University, St. Louis, MO, USA; 3 University of Washington, Seattle, WA, USA

**Keywords:** Sexual minorities, Health-related disparities, Substance use, Depressive symptoms, CRP

## Abstract

**Background:**

Growing evidence documents elevated behavioral risk among sexual-minorities, including gay, lesbian, and bisexual individuals; however, tests of biological or psychological indicators of stress as explanations for these disparities have not been conducted.

**Methods:**

Data were from the 2005-2010 National Health and Nutrition Examination Survey, and included 9662 participants; 9254 heterosexuals, 153 gays/lesbians and 255 bisexuals. Associations between sexual orientation and tobacco, alcohol, substance, and marijuana use, and body mass index, were tested using the chi-square test. Stress, operationalized as depressive symptoms and elevated C-reactive protein, was tested as mediating the association between sexual orientation and behavioral health risks. Multiple logistic regression was used to test for mediation effects, and the Sobel test was used to evaluate the statistical significance of the meditating effect.

**Results:**

Gays/lesbians and bisexuals were more likely to report current smoking (*p* < .001), a lifetime history of substance use (*p* < .001), a lifetime history of marijuana use (*p* < .001), and a lifetime period of risky drinking (*p* = .0061). The largest disparities were observed among bisexuals. Depressive symptoms partially mediated the association between sexual orientation and current smoking (aOR 2.04, 95% CI 1.59, 2.63), lifetime history of substance use (aOR 3.30 95% CI 2.20, 4.96), and lifetime history of marijuana use (aOR 2.90, 95% CI 2.02, 4.16), among bisexuals only. C-reactive protein did not mediate the sexual orientation/behavior relationship.

**Conclusion:**

Higher prevalence of current smoking and lifetime history of substance use was observed among sexual minorities compared to heterosexuals. Among bisexuals, depressive symptoms accounted for only 0.9-3% of the reduction in the association between sexual orientation and marijuana use and tobacco use, respectively. More comprehensive assessments of stress are needed to inform explanations of the disparities in behavioral risk observed among sexual minorities.

## Background

Sexual minorities, including lesbian, gay, and bisexual (LGB) individuals, have elevated behavioral risks, relative to heterosexuals. A larger proportion of LGB’s report current tobacco use (24-37%) [1], marijuana use (23-25%) [2,3], substance use (17%-30%), and risky drinking (17-25%) [4], compared to heterosexuals. Among sexual minority women, a larger proportion of obesity (34%) and overweight (30-35%) has also been documented [5], as well as elevated cardiovascular disease risk [6,7], relative to heterosexual women.

There have been few published empirical tests of the mechanisms for these documented disparities among sexual minorities. Reducing and eliminating disparities first requires identifying and testing the factors that explain the disparities observed among sexual minorities. One accepted theoretical perspective is that chronic social stressors are a root cause of the disparities observed among sexual minorities. According to the Theory of Minority Stress, sexual minorities face chronic and institutionalized distal and proximal stressors. Distal stressors take the form of stigma, prejudice, and discrimination due to living outside the heterosexual majority, and proximal stressors include internalized homophobia, expectation of rejection, and concealment of one’s sexual orientation [8]. Proximal and distal minority stressors are chronic in that they are ever present and institutional in that they are produced and promoted by social structures and institutions beyond the control of individuals. Minority stressors are cumulative and occur in addition to the general stressors associated with daily living and life events, suggesting that sexual minorities may have more exposure to stressors than heterosexuals, and may be more susceptible to stress-sensitive behavioral risks. Published results from empirical tests conducted with general stress theories, indicate that exposure to stressors alters individuals physiology and how individuals think, feel, and behave [9,10]. The chronic and cumulative nature of minority stressors may be a key driver in behavioral risk disparities identified among sexual minorities. Exposure to minority stress may promote risky behavioral coping strategies involving tobacco, alcohol, and substance use, as well as disparate rates of obesity or overweight among sexual minority women. Little empirical evidence exists to support the hypothesis that stress is involved in explaining the differences in risk behaviors. Therefore, it is important to test stress as a mediator that may explain the association between sexual orientation and risk behavior.

There are many ways to measure stress. The experience of stress is a multicomponent process of dysregulation that can be measured with both psychological and biological assessments [11]. We have conceptualized depressive symptoms and plasma C-reactive protein (CRP) as multiple markers of stress experienced by sexual minorities [12,13]. Depressive symptoms are an indicator of the psychological dysregulation resulting from exposure to minority stress among sexual minorities. Depressive symptoms manifest as diminished pleasure in activities, feeling down or hopeless, trouble sleeping, having little energy, poor appetite, and/or feeling bad about oneself [14]. CRP is a biological indicator of dysregulation in the form of inflammation [15] which has been linked to acute and chronic psychosocial stressors [16-18]. Therefore elevated CRP could indicate exposure to chronic and acute psychosocial stressors, such as minority stress, among sexual minorities.

Our study had two aims. First we aimed to confirm previous evidence of behavioral risk disparities by sexual orientation. We hypothesized that sexual minorities would have greater behavioral health risks compared to heterosexuals. Second, we tested psychological and biological markers of stress as mediators to explain the association between sexual orientation and behavioral health risks. We hypothesized that both depressive symptoms and CRP would mediate the relationship between sexual orientation and health behavior risks.

## Methods

### Study participants

Data for this study were publically available from the National Health and Nutrition Examination Survey (NHANES), pooled from 2005-2010. The NHANES is a national health surveillance program that uses in-home interviews and physical exams to assess the health and nutrition of adults and children living in the United States. NHANES is an annual cross-sectional survey of a nationally representative sample of approximately 5,000 individuals. This design is advantageous for studying small population groups, as data can be combined across years to provide adequate sample size. More detailed information regarding the NHANES design and sampling strategies are described elsewhere [19]. Data from 2005-2010 were selected for the current study, as, prior to 2005, only a subset of respondents were assessed for the presence of depressive symptoms using a different assessment than the Patient Health Questionnaire (PHQ-9).

From 2005-2010, there were 31,039 participants in the NHANES. Of these participants, only 9,929 were administered the sexual behavior questionnaire and had data available in the public use data set. The current study was based on a sample of 9,662 participants who answered the sexual orientation question and provided a response of heterosexual, gay, lesbian or bisexual. Of these participants, 9,254 were heterosexually identified, 153 identified as gay/lesbian, and 255 identified as bisexual. Those who identified their sexual orientation as “something else” (n = 57), “not sure” (n = 116), and “don’t know” (n = 61) were excluded because we could not definitively infer the meaning of their sexual orientation. An additional 33 participants were excluded because they refused to provide an answer for the sexual orientation question.

### Measures

#### Dependent variables

Health-related dependent variables included tobacco use, lifetime history of drug use, lifetime history of marijuana use, past year risky drinking, lifetime period of risky drinking, and body mass index (BMI). Participants were classified as ‘current smokers’ if they answered either “Some days” or “Every day” to the question, “Do you now smoke cigarettes?” Lifetime history of drug use, excluding marijuana, was measured with a single question, “Have you ever used cocaine, crack cocaine, heroin, or methamphetamine?”, and lifetime history of marijuana use was measured with a single question, “Have you ever, even once, used marijuana or hashish?” Past year risky drinking was assessed with the question, “In the past 12 months, on how many days did you have 5 or more drinks of any alcoholic beverage?”, and lifetime risky drinking was assessed with the question, “Was there ever a time or times in your life when you drank 5 or more drinks of any kind of alcoholic beverage almost every day?” Responses for substance use variables were dichotomized. BMI was defined as weight in kilograms divided by height in meters squared. BMI values 25.0-29.9 were considered overweight, and values greater than or equal to 30 were considered obese. These BMI categories were combined to form a single overweight/obese category.

#### Independent variables

##### Sexual orientation

Participant’s sexual orientation was assessed using the question: “Do you think of yourself as …Heterosexual or straight (that is, sexually attracted only to men); homosexual or lesbian (that is, sexually attracted only to women); bisexual (that is, sexually attracted to men and women); something else; or you're not sure?” Participants were categorized as either, Heterosexual, Lesbian/Gay or Bisexual for the purposes of analysis.

##### Mediators

Depressive symptoms and elevated CRP level served as mediators in this study. Depressive symptoms were assessed with the Patient Health Questionnaire (PHQ-9) with scores greater than or equal to10 considered moderate/greater depression and those scores below 10 considered low/no depression [14]. Serum CRP levels were measured using latex-enhanced nephelometry and classified as elevated if they were greater than 3.0 mg/L based on the recommendation of American Heart Association (AHA) and the Centers for Disease Control and Prevention (CDC) for identifying persons at increased risk for cardiovascular [20]. Details regarding laboratory procedures are described elsewhere [21].

### Demographic variables

In addition to participant’s health-related outcomes and sexual orientation, respondent’s age, gender, and education were reported and included in the analyses. Age was recoded into four categories (less than 29, 30-39, 40-49, and 50 or older), education was recoded into four categories (less than high school, high school, some college, college graduate or above) and income was recoded into five categories (less than $25k, $25k to 34, 999, $35k to $44,999, $45k to $54,999 and $55k or greater).

### Statistical analysis

Descriptive and summary statistics were calculated to describe the sample’s demographic characteristics. The Rao-Scott chi-square test for weighted analysis (a design-adjusted version of the Pearson chi-square test) was used to test for associations between sexual orientation and demographic characteristics. Associations between sexual orientation and tobacco, alcohol, and substance use, and BMI were also tested using the Rao-Scott chi-square test for weighted analysis. Evidence for mediation requires the satisfaction of four criteria [22,23]. The first two criteria require that a significant association between the independent variable and the outcome variable (criteria 1) and the independent variable and the mediator (criteria 2) be established. We evaluated these criteria using logistic regression to examine the association between sexual orientation and each behavioral risk factor (criteria 1) and the association between sexual orientation and depressive symptoms and elevated CRP (criteria 2). The third criterion requires that the mediator be significantly associated with the outcome variable. We evaluated this criterion using multiple logistic regression to examine the association between each mediator and behavioral risk factor, controlling for sexual orientation. According to Baron and Kenny’s [22] criteria, if any of the first three criteria are not satisfied, the mediation analysis will be terminated. The final criterion requires assessing the degree of attenuation between the independent and outcome variables after adjustment for the mediator. For this criterion, we utilized multiple logistic regression to evaluate the association between sexual orientation and each behavioral risk factor before and after adjustment for the mediator. To test for mediation among sexual minorities, heterosexual sexual orientation was used as referent group for all mediation analyses. The heterosexual group was selected as the referent group because it permitted comparing behavioral risk among sexual minorities to the majority group and to test stress as a possible mediator of these disparities. The Sobel test was utilized to evaluate the statistical significance of the meditating effect [24]. The Sobel test was selected to evaluate the significance of the mediating effect because of its conservative estimates [25] and ability to incorporate weights for complex sampling designs. Separate mediation analyses were conducted for gays/lesbians and bisexuals, and all models controlled for gender, age, and education. Education has shown to be a strong indicator of socioeconomic status [26]. Education may also be advantageous to income as a control variable because educational attainment is more likely reported by the majority of respondents, where not all respondents have or are willing to report income [27]. For these reasons we have elected to use education as an adjustment variable for socioeconomic status. All analyses were conducted with SAS 9.3 [28] incorporating both the design information and weights as specified by the NHANES Analytic Reporting Guidelines [29].

## Results

Table 1 presents the sample’s demographic characteristics and the prevalence of behavioral health risks by sexual orientation. Significant differences were found in regards to gender, age, education and income. Heterosexual males and females were equally represented in the sample. However, among gays/lesbian a larger proportion was male (64%) than female (32%), and the opposite was true for bisexuals, where a larger proportion was female (74%) than male (26%) (*χ*^2^ = 31.6, *p* < .001). Bisexuals tended to be younger than either heterosexuals or gays/lesbians (*χ*^2^ = 26.7, *p* < .001). More gays/lesbians (47%) than heterosexuals (27.9%) or bisexuals (19.6%) reported college graduation or higher education (*χ*^2^ = 30.2, *p* < .001). Current smoking (*χ*^2^ = 25.9, *p* < .001), lifetime history of drug use (excluding marijuana) (*χ*^2^ = 36.0, *p* < .001), lifetime history of marijuana use (*χ*^2^ = 37.4, *p* < .001), and lifetime period of risky drinking (*χ*^2^ = 10.2, *p* = .0061), varied by sexual orientation. Compared to heterosexuals, more gays/lesbians and bisexuals reported current smoking, lifetime history of drug use, lifetime history of marijuana use, and lifetime period of risky drinking. Compared to all groups, bisexuals reported the largest percentage of current smoking (41.4%), lifetime history of drug use (41.3%), lifetime history of marijuana use (79.7%), and lifetime period of risky drinking (17.5%). There was no association between high CRP and sexual orientation. Compared to all groups, bisexuals reported the largest percentage of current depressive symptoms (8.8%) (*χ*^2^ = 26.8, *p* < .001).

**Table 1:** Prevalence of demographic characteristics, behavioral risk factors and mediating variables by sexual orientation

	**Heterosexual (N = 9254)**	**Gay/Lesbian (N = 153)**	**Bisexual (N = 255)**	
		**%**		p-value*
**Demographic Characteristic**				
Gender				<0.001
Male	50.6%	63.7%	26.0%	
Female	49.4%	32.3%	74.0%	
Age Category				<0.001
Less than 29	25.7%	22.8%	39.8%	
30-39	23.5%	29.3%	26.9%	
40-49	26.4%	30.3%	17.6%	
50 or greater	23.9%	17.7%	15.7%	
Race				0.1083
Non-Hispanic White	68.4%	70.5%	70.2%	
Non-Hispanic Black	11.5%	11.9%	16.4%	
Mexican American	9.3%	6.9%	5.9%	
Other Hispanic	4.9%	4.4%	4.0%	
Other (Including Multi-Racial)	5.9%	6.3%	3.5%	
Education				<0.001
Less than High School	15.8%	9.5%	17.8%	
High School	23.6%	9.2%	24.5%	
Some College	32.7%	34.2%	38.1%	
College Graduate or Above	27.9%	47.1%	19.6%	
Annual Household Income				<0.001
Less than $25,000	17.9%	20.5%	34.4%	
$25,000 - $34,999	9.6%	9.9%	9.6%	
$35,000 - $49,999	8.3%	5.5%	12.3%	
$45,000 to $54,999	9.5%	16.5%	9.7%	
$55,000 or greater	54.6%	47.7%	34.0%	
**Behavioral Risk Factors**				
Current Smoker				<0.001
Yes	25.0%	30.6%	41.4%	
BMI				0.405
Overweight/Obese	44.1%	38.7%	45.6%	
BMI				0.1894
Obese	22.6%	26.3%	27.7%	
Lifetime History of Drug Use (Excluding Marijuana)				<0.001
Yes	20.5%	30.8%	41.3%	
Lifetime History of Marijuana Use				<0.001
Yes	59.5%	72.9%	79.7%	
Risky Drinking (5 or more drinks) in Past 12 months				0.4063
Yes	23.3%	25.1%	28.1%	
Lifetime Period of Risky Drinking				0.0061
Yes	9.7%	10.5%	17.5%	
**Mediating Variables**				
RaceHigh CRP (greater than 3.0 mg/L)				0.998
Yes	24.5%	24.4%	24.7%	
Current Depressive Symptoms (PHQ-9 greater than 10)				<0.001
Yes	4.8%	4.8%	8.8%	

*p-value based on the Rao-Scott Chi-Square Chi-Square Test for Weighted Analysis.

Table 2 presents unadjusted associations between sexual orientation and behavioral health risks, depressive symptoms and elevated CRP. Depressive symptoms were not significantly associated with lesbian/gay orientation in the unadjusted analysis; however, they were significantly associated after adjustment for socio-demographic characteristics (aOR 2.38, 95% CI 1.02, 5.56). Elevated CRP was not significantly associated with lesbian/gay or bisexual sexual orientation in either unadjusted or adjusted analyses, and therefore did not meet criteria for further tests of mediation [23].

**Table 2:** Unadjusted associations between sexual orientation, behavorial risk factors and mediating variables

	**Gay/Lesbian (N = 153)**		**Bisexual (N = 255)**	
**Outcome**	OR*	95% CI**	OR*	95% CI**
	**Behavioral Risk Factors**			
Current Smoker	1.32	(0.83, 2.12)	2.12	(1.70, 2.64)
BMI (Overweight/Obese)	1.06	(0.75, 1.51)	0.80	(0.55, 1.16)
Lifetime History of Drug Use (Excluding Marijuana)	1.73	(1.10, 2.72)	2.73	(1.87, 4.00)
Lifetime History of Marijuana Use	1.83	(1.17, 2.84)	2.66	(1.89, 3.75)
Risky Drinking (5 or more drinks) in Past 12 months	1.10	(0.68, 1.80)	1.29	(0.88, 1.87)
Lifetime Period of Risky Drinking	1.09	(0.64, 1.86)	1.98	(1.22, 3.20)
	**Mediating Variables**			
Current Depressive Symptoms (PHQ-9 greater than 10)	1.94	(0.83, 4.55)	3.71	(2.12, 6.47)
High CRP (greater than 3.0 mg/L)	1.00	(0.58, 1.73)	1.01	(0.77, 1.32)

*Odds Ratio with Heterosexuals as the reference category.

**Confidence Interval.

Table 3 presents the association between sexual orientation and each behavioral risk factor controlling for socio-demographic characteristics, where heterosexual sexual orientation served as the referent group. Model 1 provides the association before adjustment for depressive symptoms, and Model 2 provides the association after adjustment for depressive symptoms. Gays/lesbians were 84% more likely than heterosexuals to report current smoking (Model 1 aOR 1.84, 95% CI 1.17, 2.92). Depressive symptoms partially mediated the association between sexual orientation and current smoking (Model 2 aOR 1.81, 95% CI 1.14, 2.88) and reduced the association by 1.68%. This finding approached but did not achieve the .05 level of significance (*p* = .06). Depressive symptoms did not mediate associations between sexual orientation and the remaining behavioral risks among gays/lesbians. Depressive symptoms were not associated with BMI, risky drinking in the past year, and lifetime risky drinking for the lesbian/gay group and the mediation analysis was terminated. Bisexuals were more than twice as likely as heterosexuals to report current smoking (Model 2 aOR 2.12, 95% CI 1.65, 2.73) (Table 3). Among bisexuals, depressive symptoms partially mediated the association between sexual orientation and current smoking (Model 3 aOR 2.04, 95% CI 1.59, 2.63), and accounted for 3.7%, of this association (*p* = .002). Bisexuals were more than three times as likely to report lifetime history of drug use (excluding marijuana) compared to heterosexuals (Model 2 aOR 3.38 95% CI 2.24, 5.10). Depressive symptoms partially mediated (Model 3 aOR 3.30 95% CI 2.20, 4.96), and accounted for 2.37% of this association (*p* = .05). Bisexuals were nearly three times as likely as heterosexuals to report lifetime history of marijuana use (Model 2 aOR 2.92, 95% CI 2.05, 4.19). Depressive symptoms partially mediated (Model 3 aOR 2.90, 95% CI 2.02, 4.16), and accounted for .92% of this association (*p* < .001). Depressive symptoms were not associated with BMI among bisexuals and mediation analysis was terminated. Tests for mediation could not be calculated for bisexual orientation and past year risky drinking or lifetime risky drinking due to sample size constraints.

**Table 3:** Associations between sexual orientation and behavioral risk factor accounting for the presence of depressive symptoms in the National Health and Nutrition Examination Survey, 2005-2010

**Gay/Lesbian**							
	**Model 1 (adjusted for socio-demographic characteristics)**^**a**^		**Model 2 (controlling for depressive symptoms)**^**b**^		**% reduction in association controlling for PHQ-9**	**Sobel test for significance of mediator**	
Outcome	OR*	(95% CI)	OR*	(95% CI)		Z Value	P-Value
Current Smoker	1.843	(1.17, 2.92)	1.812	(1.14, 2.88)	1.68	1.846	0.064
BMI (Overweight/Obese)	1.128	(0.78, 1.64)					
Lifetime History of Drug Use (Excluding Marijuana)	1.786	(1.10, 2.91)	1.766	(1.09, 2.87)	1.12	1.4863	0.137
Lifetime History of Marijuana Use	1.753	(1.09, 2.82)	1.746	(1.09, 2.81)	0.40	1.50534	0.132
Risky Drinking (5 or more drinks) in Past 12 months	1.01	(0.61, 1.69)					
Lifetime Period of Risky Drinking	1.271	(0.76, 2.12)					
Bisexual							
	**Model 1 (adjusted for socio-demographic characteristics)**^**a**^		**Model 2 (controlling for depressive symptoms)**^**b**^		**% reduction in association controlling for PHQ-9**	**Sobel test for significance of mediator**	
Outcome	OR*	(95% CI)	OR*	(95% CI)		Z Value	P-Value
Current Smoker	2.124	(1.65, 2.73)	2.044	(1.59, 2.63)	3.77	3.04054	0.0023
BMI (Overweight/Obese)	0.897	(0.61, 1.31)		-	-	-	-
Lifetime History of Drug Use (Excluding Marijuana)	3.381	(2.24, 5.10)	3.301	(2.20, 4.96)	2.37	1.93298	0.0532
Lifetime History of Marijuana Use	2.928	(2.05, 4.19)	2.901	(2.02, 4.16)	0.92	3.44351	<0.001
Risky Drinking (5 or more drinks) in Past 12 months	1.499	(1.05, 2.15)	Cannot Estimate				
Lifetime Period of Risky Drinking	2.901	(1.67, 5.04)	Cannot Estimate				

^a^adjusted for gender, age and education.

^b^adjusted for gender, age, education and presence of moderate or greater depressive symptoms.

*compared to heterosexuals as referent category.

-mediator not associated with the dependent variables.

## Discussion

The first aim of this study was to confirm the existence of disparities in behavioral health risks among sexual minorities. Our findings confirm that gays/lesbians and bisexuals had a greater prevalence of current smoking, and lifetime history of substance use compared to heterosexuals. The prevalence rates of current smoking and lifetime history of substance use among gays/lesbians in our sample were similar to the rates reported by others [2,4,30], and the prevalence rate of lifetime history of marijuana use among gays/lesbians (59%) was more than double the prevalence published in previous reports (25%) [3]. The difference in prevalence of marijuana use may result from differences in measurement between studies. Previous studies asked respondents how often they used marijuana in the past year [3], where respondents in our sample reported on their lifetime use of marijuana.

Bisexuals had the highest prevalence of current smoking, lifetime substance and marijuana use, and risky drinking, compared to both gay/lesbian and heterosexual subgroups. These finding are similar to the results published by others who have reported increased tobacco, alcohol and substance use among bisexual individuals [3,4,31], compared to gays/lesbians and heterosexuals [4,32]. The disparities in behavioral risks identified among bisexuals suggest that bisexual individuals may be at greater risk than both gays/lesbians and heterosexuals for future chronic health problems related to tobacco, alcohol, and substance use.

The second aim of this study was to test depressive symptoms and CRP as possible mediators of the relationship between sexual orientation and behavioral risks. Depressive symptoms partially explained current smoking, lifetime marijuana use and lifetime substance use, but only among bisexuals. Depressive symptoms accounted for 3% of the reduction in the association between sexual orientation and current smoking among bisexuals, and even less in lifetime substance use (2%) and in lifetime marijuana use (.9%). The association between sexual orientation and smoking and substance use was very robust, and while depressive symptoms lessened the association, it is apparent from the small percentage of the association attributable to depressive symptoms, that there may be other key drivers contributing to the behavioral risk disparities observed among sexual minorities or that depressive symptoms were not sensitive enough indictors of stress. Conducting comprehensive assessments of minority stress, mental health, and coping could inform explanations of the disparities in health-related outcomes observed among sexual minorities.

Elevated CRP was not associated with sexual orientation. Few sexual minorities in this sample had elevated CRP, making an association difficult to detect. Larger samples of sexual minority participants could help to inform this issue. Further it is possible that a single biomarker of stress is not adequate to represent the physiological effects of minority stress among sexual minorities.

This study has several limitations. The data were cross-sectional and this restricted our ability to test causal relationships between the variables of interest that may unfold over time. Additionally, comprehensive measures of minority stress were unavailable for testing and depressive symptoms were used as a proxy to indicate stress. What is known about the role of minority stress and behavioral health risks among sexual minorities could be significantly improved with the future use of longitudinal methodologies and comprehensive assessments of stress with a cohort of sexual minorities. Comprehensive assessments of stress should include measures of environmental demands such as discrimination, cognitive and psychological responses including measures of internalized homophobia and stigma, and anxiety, as well as biological indictors of the stress process including cortisol. A more thorough assessment could contribute much needed evidence concerning the psychological and physiological experience of stress as it relates to behavioral risk among sexual minorities. Finally, the sample sizes of sexual minority subgroups were relatively small and limited our ability to conduct mediation analyses of risky drinking among bisexual individuals. Small sample size also limited further subgroup analyses (e.g., race/ethnicity). Larger samples of sexual minority respondents would allow for these tests. In the future, oversampling sexual minorities in large health surveillance programs could resolve this problem.

This study has notable strengths including its national, population-based, sample. This study fills a gap in the current literature by empirically testing depressive symptoms as a possible explanation of disparities in behavioral risks. This is relevant because the most prominent theoretical explanations for health-related disparities among sexual minorities point to symptoms of stress, such as depressive symptoms, as drivers in health disparities, and yet there is a paucity of publications that provide empirical tests of the factors that drive disparities. This work is also responsive to national calls for empirical investigations that use population-based methods to confirm and test explanations for health-related disparities identified among sexual minority populations [33]. Our study fills these needs.

## Conclusions

The findings presented by this report provide initial support for the idea that stress, as represented in this study by depressive symptoms, may be a driver in health-related disparities among sexual minorities. Although indicators of stress did not entirely explain the observed disparities in behavioral risks among sexual minorities, it was linked to decreased risk among bisexual individuals, the highest risk group. In context of the growing evidence of elevated behavioral risk among sexual minorities, our study has provided indications that chronic stress may be involved in some behavioral disparities among sexual minorities, but there is an urgent need to test comprehensive measures of stress, including biological indices, and indicators of minority stress, as mediators of these disparities, if we are to better understand the factors driving behavioral disparities among sexual minorities. Such evidence is necessary for developing and implementing successful multi-level interventions, in the form of policy, community-based programming, and behavioral interventions, which can eliminate disparities.

## Competing interests

The authors declare that they have no competing interests.

## Authors’ contributions

JJ GF DB collaboratively conceptualized and designed this study. JJ drafted the manuscript, interpreted results, and contributed to editorial needs. GF conducted statistical analyses, contributed substantively to the methods, and provided feedback and editorial support. DB contributed conceptual feedback regarding interpretations, and provided significant support to manuscript revision and editorial needs. All authors read and approved the manuscript.

## Pre-publication history

The pre-publication history for this paper can be accessed here:

http://www.biomedcentral.com/1471-2458/14/401/prepub
